# Trends in mortality, pathogen distribution, and antimicrobial resistance of peritoneal and intra-abdominal infections from 1990 to 2021: a cross-sectional analysis of the MICROBE database

**DOI:** 10.1097/JS9.0000000000004587

**Published:** 2025-12-18

**Authors:** Qiancheng Xu, Yan Qian, Mengqi Guan, Min Liu, Shuoshuo Wei, Yingya Cao, Xiaogan Jiang, Weihua Lu, Lei Zha

**Affiliations:** a Department of Critical Care Medicine, The First Affiliated Hospital of Wannan Medical College, Yijishan Hospital of Wannan Medical College, Wuhu, Anhui, China; b Anhui Provincial Clinical Research Center for Critical Respiratory Disease, Wuhu, Anhui, China; c Perioperative Monitoring and Prognostic Technology Research and Development Center of Wuhu, Wuhu, Anhui, China; d Department of Emergency Intensive Care Unit, Wuhu Hospital, East China Normal University, Wuhu, Anhui, China; e Department of Pulmonary and Critical Care Medicine, Wuhu Hospital, East China Normal University, Wuhu, Anhui, China; f Department of Respiratory Medicine, The First Affiliated Hospital of Wannan Medical College, Yijishan Hospital of Wannan Medical College, Wuhu, Anhui, China

**Keywords:** antimicrobial resistance, epidemiology, global burden, intra-abdominal infections, pathogen distribution

## Abstract

**Background:**

Peritoneal and intra-abdominal infections (IAIs) are a major cause of morbidity and mortality in surgical patients, with rising antimicrobial resistance (AMR) threatening surgical safety. This study mapped global and regional patterns in IAI burden and resistant pathogens to inform evidence-based surgical practice.

**Methods:**

Global IAI deaths, disability-adjusted life years, and distributions of key AMR pathogens were analyzed from 1990 to 2021 across 21 Global Burden of Disease regions using the Measuring Infectious Causes and Resistance Outcomes for Burden Estimation (MICROBE) database, with future projections through 2050.

**Results:**

IAI-related deaths rose from 394 500 [95% uncertainty intervals (UIs) 361 200–427 800] in 1990 to 646 359 (95% UIs 585 349–707 369) in 2021, with mortality rates increasing from 7.4 (95% UIs 6.8–8.0) to 8.2 (95% UIs 7.4–9.0) per 100 000 population. By 2050, global deaths are projected to decrease slightly by 0.6% to 642 485 (95% UIs 552 824–750 367). However, this decrease is not seen in all regions. The death count is predicted to rise in Latin America and the Caribbean, while substantial declines are projected for South Asia, Central/Eastern Europe, and Central Asia. *Escherichia coli* was the leading global pathogen, with regional variation: *Klebsiella pneumoniae* predominated in sub-Saharan Africa, while *Staphylococcus aureus* was most frequent in high-income regions. In 2021, AMR-related deaths approached 329 000 worldwide, with the death rate rising from 3.79 to 4.17 per 100 000. Carbapenem-resistant *Acinetobacter baumannii* and *Klebsiella pneumoniae* showed the most rapid increases. Methicillin-resistant *S. aureus* mortality remained stable, while efforts to control vancomycin-resistant *Enterococcus faecium* were less effective.

**Conclusion:**

The rising and regionally diverse burden of IAIs, exacerbated by increasing AMR, demands robust surgical antimicrobial stewardship and targeted infection control strategies. Tailoring perioperative antibiotics to local resistance patterns and prioritizing effective source control are essential to optimizing surgical outcomes worldwide.

## Introduction

Peritoneal and intra-abdominal infections (IAIs) impose a considerable global burden, leading to high morbidity and placing substantial demands on healthcare systems[1]. A recent analysis of the global burden of bacterial infections estimated that IAIs caused approximately 1.28 million deaths worldwide in 2019[2]. Despite this alarming impact, significant gaps remain in our understanding of how IAI burden, etiologic pathogens, and antimicrobial resistance (AMR) patterns vary across regions and evolve over time. We hypothesized that the global mortality burden from IAIs has significantly increased between 1990 and 2021, driven predominantly by the emergence of AMR pathogens, with substantial heterogeneity across Global Burden of Disease (GBD) regions.

Previous multi-center studies have consistently identified IAIs, often arising as postoperative complications or requiring urgent surgical intervention, as a leading source of infection in intensive care units and a major driver of mortality^[3–5]^. For instance, landmark audits like ICON, EPIC II, and EPIC III revealed that the abdomen is the second most common site of infection in critically ill patients, following the respiratory tract^[5–7]^. However, these studies, despite being invaluable, often provide cross-sectional snapshots primarily from high-resource settings. Consequently, a comprehensive longitudinal analysis of IAIs across varied geographic and economic contexts is lacking. This limitation is particularly critical as mortality rates for IAIs have not significantly declined in recent decades, despite advances in clinical care[8], suggesting that region-specific challenges, such as pathogen distribution and AMR, may be hampering progress and underscoring the need to understand how these factors intersect with surgical practices and source control strategies. Unlike broader intra-abdominal sepsis, which encompasses diverse conditions with heterogeneous etiologies and management pathways, IAIs represent a more clinically and surgically defined entity. This focus allows for more precise linkage between microbiological profiles, AMR trends, and operative interventions. Such information is directly actionable for perioperative decision-making and infection control programs. In addition, prior AMR burden studies have often aggregated abdominal infections into broader sepsis categories. This can obscure procedure-related risks and pathogen-specific resistance patterns. Isolating IAIs as a distinct analytic unit provides the level of detail necessary to inform surgical prophylaxis protocols and optimize empiric antimicrobial choices.

The rising prevalence of multidrug-resistant organisms (MDROs), including extended-spectrum β-lactamase-producing Enterobacterales, carbapenem-resistant *Klebsiella pneumoniae* (CRKP), and multidrug-resistant *Acinetobacter baumannii*, has profoundly complicated the clinical and surgical management of IAIs^[9,10]^. This surge in resistance directly challenges the efficacy of perioperative antibiotic prophylaxis and empirical treatment regimens, thereby limiting therapeutic options, increasing healthcare costs, and worsening patient outcomes^[2,11]^. Addressing this global health threat requires robust, standardized data to inform targeted interventions. Yet, large-scale studies detailing the long-term trends of specific pathogens and their resistance profiles in IAIs at both global and regional levels remain scarce. We hypothesized that global IAI mortality has increased over three decades, disproportionately affecting older adults, and that AMR substantially contributes to this burden.

To address this critical knowledge gap, our study provides a comprehensive analysis of IAI trends from 1990 to 2021 by leveraging the Measuring Infectious Causes and Resistance Outcomes for Burden Estimation (MICROBE) database, which is an integral component of the highly influential GBD study^[12,13]^. This research, to our knowledge, represents the first study to systematically investigate the global and regional burden of IAIs at the pathogen and AMR levels over a three-decade period. By analyzing data across 21 GBD regions, we aim to elucidate long-term trends in IAI-related mortality, pathogen distribution, and AMR prevalence, and further project their future trajectories from 2022 to 2050 using predictive modeling. The findings from this study are intended to provide critical evidence to guide global surveillance efforts, refine regional treatment strategies, including perioperative antibiotic guidelines, and support the development of data-driven public health policies to mitigate the impact of these deadly infections. This cross-sectional study was conducted and reported in strict accordance with the STROCSS (Strengthening the Reporting of Cohort Studies in Surgery) guidelines[14]. Furthermore, all phases of research design and manuscript preparation rigorously complied with the TITAN (Transparency In The reporting of Artificial INtelligence) Guidelines 2025[15].

**HIGHLIGHTS**

- Global intra-abdominal infections (IAIs) deaths surged from 394 500 [95% uncertainty intervals (UIs) 361 200–427 800] to 646 359 (95% UIs: 585 349–707 369) (1990−2021) and are projected to remain high to 2050, with starkly divergent regional trajectories.
- Pathogen distribution varies by region, challenging “one-size-fits-all” prophylaxis: *Escherichia coli* predominates globally, carbapenem-resistant *Klebsiella pneumoniae* (CRKP) hotspots in North Africa and the Middle East, while *Staphylococcus aureus* is a key target in high-income regions.
- Mortality attributable to antimicrobial resistance (AMR) has risen substantially, driven primarily by CRKP and carbapenem-resistant *Acinetobacter baumannii*.
- The dual burden of high IAI mortality and escalating AMR underscores the urgent need for tailored surgical stewardship, enhanced infection prevention, and prioritizing effective source control.

## Methods

### Study design and data sources

We performed a systematic analysis using data from the MICROBE database (updated on 16 September 2024 and accessed on 10 December 2024), maintained by the Institute for Health Metrics and Evaluation (IHME) at the University of Washington. The database is openly accessible at https://vizhub.healthdata.org/microbe/, under a non-commercial license, requiring users to register and agree to its terms before download. Comprehensive details about MICROBE’s methodological framework have been published previously^[2,12,13,16]^. Comprehensive details regarding the modeling methodology, data acquisition, and approaches for handling regions with limited data can be found in the Methods section of the Supplemental Digital Content File 1, available at: http://links.lww.com/JS9/G517. As the MICROBE database relies on statistical modeling to combine heterogeneous surveillance and literature sources, all estimates carry inherent limitations related to the variability of primary data coverage, diagnostic practices, and reporting completeness across regions and over time. These factors are addressed through standardization, bias adjustments, and covariate-based extrapolation, but may result in wider uncertainty intervals (UIs) in regions with sparse data.

### Data extraction and variables

MICROBE contains estimates of mortality and disability-adjusted life years (DALYs) for various infectious syndromes and corresponding pathogen–drug combinations. The dataset spans numerous countries and territories and provides both “AMR-associated burden” and “AMR-attributable burden.” In this study, *AMR-associated burden* refers to the total mortality or DALYs in which antimicrobial-resistant infections were present, compared to a counterfactual scenario in which no infection occurred. AMR-attributable burden isolates the excess mortality or DALYs directly caused by resistant pathogens, compared to a counterfactual in which infections were only due to susceptible strains.

### Handling variability in diagnostic capacity and reporting

We applied the MICROBE estimation framework to address differences in diagnostic coverage, laboratory methods, and reporting accuracy across countries. The methodological framework is designed to ensure the robustness of these estimates through rigorous data validation, including the standardization of definitions and harmonization of susceptibility data, and to mitigate the effects of key confounders, such as underlying patient severity, via the attributable burden counterfactual. Point estimates were generated from repeated posterior draws, with 95% UIs defined by the 2.5th and 97.5th percentiles. Specifically, hierarchical modeling with nested random effects was used, incorporating the Healthcare Access and Quality Index as a covariate to capture regional disparities in diagnostic and treatment infrastructure. Crosswalking adjustments harmonized pathogen and resistance prevalence data derived from differing microbiological protocols. For countries or regions lacking routine surveillance, estimates were informed by structured evidence synthesis, including systematic reviews and neighboring-country extrapolations. These steps help reduce bias and improve comparability, while maintaining transparency about the uncertainty inherent in extrapolated estimates. For detailed information regarding the estimation process, refer to Supplemental Digital Content File 1, available at: http://links.lww.com/JS9/G517. For this study, we focused on data pertaining to IAIs over the period 1990–2021. We extracted global and regional estimates of deaths and DALYs, placing particular emphasis on infections involving MDRO, including carbapenem-resistant strains. The analytical output was organized according to the GBD classification scheme, which encompasses 21 regions and 7 super-regions. A full listing of countries within these categories can be found in earlier IHME documentation and Supplemental Digital Content Table S1, available at: http://links.lww.com/JS9/G517^[2,12,13,16]^.

### Analytical approach

We collated all relevant data on mortality and DALYs attributable to IAIs from the GBD 2021 study. To evaluate future trends, we utilized a Bayesian age–period–cohort (BAPC) model to forecast the disease burden from 2022 to 2050^[12,13,16]^. Detailed description of the BAPC method principle is provided in Supplemental Digital Content File 2, available at: http://links.lww.com/JS9/G517. Our analysis also highlighted the contributions of specific resistant pathogens where data were available. All estimates are presented with their corresponding 95% UIs to quantify statistical uncertainty. Narrow UIs indicate greater precision and reliability of trends, whereas wide UIs reflect higher uncertainty, often due to sparse data or variability in surveillance quality. Annual percentage change (APC) estimates with UIs overlapping zero were considered indicative of no statistically significant change over time.

### Ethical considerations

All information used in this analysis was derived from secondary sources without direct patient identifiers. As data from the MICROBE database are publicly available (subject to a non-commercial use license), no additional ethical review was required for this study.

## Results

### Burden of IAIs

Globally in 2021, IAIs ranked fifth among all infection-related causes of death worldwide, accounting for 5.51% of total global infection-related deaths, and have exhibited an overall upward trend over the past three decades (Supplemental Digital Content Figure S1, available at: http://links.lww.com/JS9/G517). Compared with 1990, the absolute number of deaths attributable to these infections increased from 394 500 (95% UIs 361 200–427 800) to 646 359 (585 349–707 369) in 2021, with the corresponding crude mortality rate rising from 7.4 (95% UIs 6.8–8.0) to 8.2 (7.4–9.0) per 100 000 population (Table 1). Notably, the highest absolute number of IAIs-related deaths in 2021 was observed in China (98 602; 95% UIs 75 963–121 242), followed by India (79 950; 65 415–94 485) and the United States (43 188; 38 772–47 605) (Supplemental Digital Content Figure S2, available at: http://links.lww.com/JS9/G517). At the same time, several countries recorded exceptionally high mortality rates per 100 000 population, led by Lithuania (25.57; 22.15–29.00) and Monaco (25.57; 20.70–30.43), with Latvia, Portugal, Japan, Russia, Spain, Hungary, and Bulgaria also exceeding 21.0 per 100 000 (Supplemental Digital Content Figure S3, available at: http://links.lww.com/JS9/G517). A log-linear regression showed an APC of approximately +0.33% in global deaths between 1990 and 2021. Regionally, the High-income Asia Pacific experienced the fastest rise, with an APC of +2.38%. Mortality also increased in Southeast Asia, East Asia, and Oceania (+1.13%), Central Europe, Eastern Europe, and Central Asia (+0.96%), and Latin America and the Caribbean (+1.24%), but decreased in South Asia (by −0.56%) and sub-Saharan Africa (by −0.62%) (Table 1). In 2021, DALYs attributable to IAIs reached 16.54 million (15.24–17.84 million), up from 11.91 million (10.99–12.83 million) in 1990. The global all-age DALYs rate declined from 223.3 (206.1–240.6) to 209.6 (193.1–226.1) per 100 000, with an APC of −0.20% (−0.49 to 0.09) (Supplemental Digital Content Table S2, available at: http://links.lww.com/JS9/G517). In some regions, despite a modest upward trend in the APC estimate, the 95% UI included zero. The finding was therefore not statistically significant and requires cautious interpretation due to variability in surveillance data.

**Table 1:** Regional trends in deaths from peritoneal and intra-abdominal infections, 1990–2021.

GBD-regions	1990		2021		APC (%)
	Number, n × 1000	Rate (per 100 000)	Number, n × 1000	Rate (per 100 000)	
Global	394.5 (361.2, 427.8)	7.4 (6.8, 8.0)	646.4 (585.3, 707.4)	8.2 (7.4, 9.0)	0.33 (−0.25, 0.90)
Southeast Asia, East Asia, and Oceania	85.8 (74.2, 97.4)	5.1 (4.4, 5.8)	154.2 (128.1, 180.3)	7.1 (5.9, 8.3)	1.13 (0.01, 2.22)
East Asia	58.5 (49.1, 67.9)	4.8 (4.0, 5.6)	103.4 (80.4, 126.4)	7.0 (5.5, 8.6)	1.26 (−0.06, 2.84)
Southeast Asia	27.1 (23.3, 30.8)	5.8 (5.0, 6.6)	50.2 (43.8, 56.7)	7.2 (6.3, 8.1)	0.73 (−0.15, 1.71)
Oceania	0.2 (0.2, 0.3)	3.6 (2.8, 4.4)	0.5 (0.4, 0.6)	3.7 (3.0, 4.4)	0.09 (−1.06, 1.59)
Central Europe, Eastern Europe, and Central Asia	51.8 (48.8, 54.8)	12.3 (11.6, 13.0)	68.8 (62.8, 74.9)	16.5 (15.0, 17.9)	0.96 (0.42, 1.48)
Central Asia	4.0 (3.7, 4.2)	5.7 (5.3, 6.1)	5.9 (5.2, 6.5)	6.1 (5.4, 6.8)	0.22 (−0.38, 1.02)
Central Europe	17.8 (16.8, 18.9)	14.3 (13.5, 15.1)	20.9 (18.9, 22.9)	18.1 (16.4, 19.9)	0.79 (0.25, 1.41)
Eastern Europe	30.0 (28.2, 31.9)	13.2 (12.4, 14.1)	42.0 (37.7, 46.3)	20.3 (18.2, 22.4)	1.45 (0.93, 2.10)
High-income	116.8 (107.9, 125.7)	12.8 (11.9, 13.8)	177.5 (155.5, 199.5)	16.3 (14.2, 18.3)	0.80 (0.24, 1.39)
High-income Asia Pacific	15.1 (13.9, 16.3)	8.7 (8.0, 9.4)	34.6 (28.8, 40.3)	18.6 (15.5, 21.7)	2.38 (1.67, 3.03)
Australasia	2.0 (1.9, 2.2)	10.0 (9.3, 10.8)	3.7 (3.3, 4.2)	12.1 (10.6, 13.5)	0.65 (0.20, 1.16)
Western Europe	62.2 (57.4, 67.0)	16.2 (14.9, 17.4)	82.7 (72.3, 93.2)	18.9 (16.5, 21.3)	0.49 (0.07, 0.96)
Southern Latin America	5.6 (5.2, 5.9)	11.3 (10.6, 12.0)	8.2 (7.5, 8.9)	12.1 (11.0, 13.1)	0.24 (−0.14, 0.63)
High-income North America	31.8 (29.2, 34.5)	11.3 (10.4, 12.3)	48.3 (43.3, 53.3)	13.0 (11.7, 14.4)	0.46 (−0.04, 0.92)
Latin America and Caribbean	27.0 (25.7, 28.3)	6.9 (6.6, 7.3)	60.5 (54.3, 66.7)	10.2 (9.1, 11.2)	1.24 (0.57, 1.86)
Caribbean	2.7 (2.5, 2.9)	7.6 (7.1, 8.2)	4.3 (3.7, 4.9)	9.0 (7.7, 10.4)	0.57 (−0.25, 1.38)
Andean Latin America	4.1 (3.6, 4.6)	10.7 (9.4, 12.1)	5.3 (4.2, 6.4)	8.0 (6.4, 9.6)	−0.94 (−1.56, − 0.33)
Central Latin America	10.6 (10.1, 11.1)	6.4 (6.1, 6.8)	27.6 (24.2, 31.0)	10.9 (9.6, 12.2)	1.74 (1.01, 2.49)
Tropical Latin America	9.6 (9.1, 10.1)	6.3 (6.0, 6.6)	23.3 (21.3, 25.3)	10.3 (9.4, 11.1)	1.60 (0.91, 2.31)
North Africa and Middle East	13.0 (11.4, 14.6)	3.8 (3.4, 4.3)	29.5 (25.7, 33.2)	4.7 (4.1, 5.3)	−0.62 (−1.61, 0.37)
South Asia	71.6 (58.5, 84.8)	6.6 (5.3, 7.8)	100.0 (85.1, 115.0)	5.4 (4.6, 6.2)	−0.56 (−1.88, 0.77)
Sub-Saharan Africa	28.5 (24.3, 32.7)	5.8 (4.9, 6.6)	56.0 (45.7, 66.2)	4.9 (4.0, 5.8)	−0.62 (−1.74, 0.49)
Central sub-Saharan Africa	2.8 (2.3, 3.3)	5.1 (4.2, 6.0)	5.9 (4.4, 7.3)	4.3 (3.2, 5.3)	1.54 (0.94, 2.18)
Eastern sub-Saharan Africa	11.6 (10.0, 13.2)	6.1 (5.2, 6.9)	21.2 (16.7, 25.6)	5.0 (3.9, 6.0)	−0.80 (−2.05, 0.49)
Southern sub-Saharan Africa	2.7 (2.3, 3.1)	5.1 (4.3, 5.9)	6.4 (5.7, 7.0)	8.0 (7.2, 8.8)	0.33 (−0.25, 0.90)
Western sub-Saharan Africa	11.4 (9.1, 13.7)	5.9 (4.7, 7.1)	22.5 (17.5, 27.6)	4.6 (3.6, 5.6)	1.13 (0.01, 2.22)

Data are presented as median with an interquartile range.

APC, annual percent change of rate.

### Projected burden of IAIs

**Figure 1.**
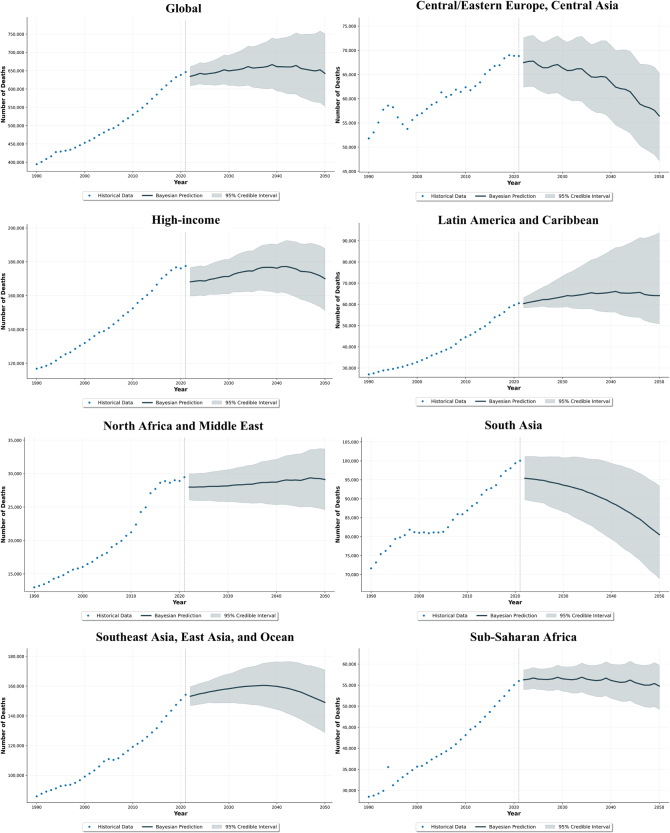
Forecasted number of deaths from intra-abdominal infections, globally and by the GBD super-region, 1990–2050. This figure presents historical (1990–2021) and projected (2022–2050) death rates (per 100 000 population) from intra-abdominal infections. Data are shown globally and for the seven GBD super-regions. Solid lines represent historical median estimates, dashed lines indicate projected medians, and shaded areas correspond to 95% UIs. In contrast to the rising absolute death toll (Fig. 1), this graph shows that the age-standardized death *rate* is projected to decline across all super-regions by 2050. This suggests that population growth and aging are major drivers of the increasing death count, and public health or clinical improvements may be having a positive impact on a per-capita basis. However, the wide UIs in several regions underscore considerable uncertainty in these projections, warranting cautious optimism.

**Figure 2.**
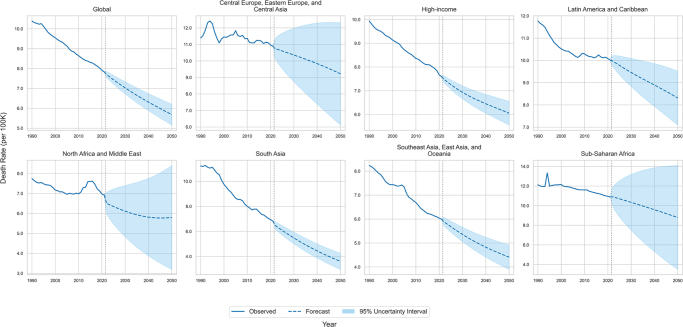
Forecasted death rate from intra-abdominal infections, globally and by the GBD super-region, 1990–2050. This figure presents historical (1990–2021) and projected (2022–2050) death rates (per 100 000 population) from intra-abdominal infections. Data are shown globally and for the seven GBD super-regions. Solid lines represent historical median estimates, dashed lines indicate projected medians, and shaded areas correspond to 95% uncertainty intervals (UIs). In contrast to the rising absolute death toll (Fig. 1), this graph shows that the age-standardized death *rate* is projected to decline across all super-regions by 2050. This suggests that population growth and aging are major drivers of the increasing death count, and public health or clinical improvements may be having a positive impact on a per-capita basis. However, the wide UIs in several regions underscore considerable uncertainty in these projections, warranting cautious optimism.

Globally, the number of deaths is forecasted to decrease slightly by 0.6%, from 646 359 (95% UIs 585 349–707 369) in 2021 to 642 485 (95% UIs 552 824–750 367) by 2050. However, this downward trend is not uniform across all regions. The absolute death count is projected to increase in Latin America and Caribbean (from 60 527 to 64 103; a 5.9% rise), while it is projected to decrease substantially in regions such as South Asia (from 100 045 to 80 522; a 19.5% decline) and Central Europe, Eastern Europe, and Central Asia (from 68 817 to 56 433; a 18.0% decline) over the same period (Fig. 1). In contrast to absolute numbers, death rates are projected to decline significantly across all GBD super-regions. The global death rate is expected to fall from 7.83 (95% UIs 7.81–7.85) to 5.68 (95% UIs 5.16–6.21) per 100 000 population between 2021 and 2050 (Fig. 2). Trends for DALYs and DALY rates were consistent with those for death numbers and death rates, respectively (Supplemental Digital Content Figures S4 and S5, available at: http://links.lww.com/JS9/G517).

### Burden of pathogen-specific IAIs

**Figure 3.**
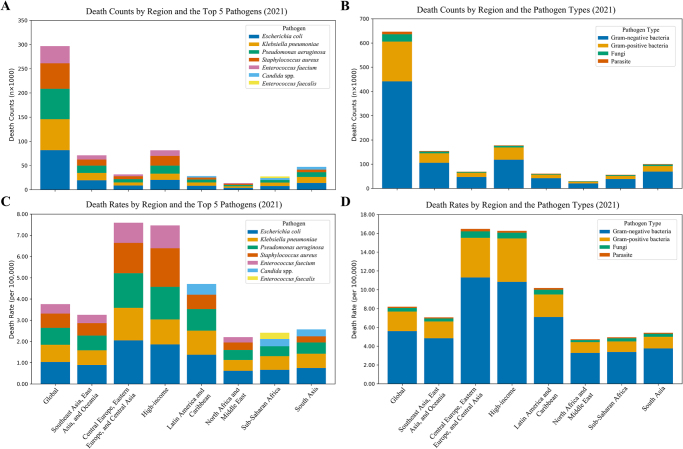
Distribution of deaths and death rates from intra-abdominal infections by pathogen and pathogen type, by GBD super-region, 2021. These panels provide a snapshot of the leading pathogens causing IAI-related mortality in 2021, broken down by the GBD super-region. Panels show (A) absolute deaths by pathogen, (B) absolute deaths by pathogen type (e.g., Gram-negative), (C) death rates by pathogen, and (D) death rates by pathogen type. All pathogen names are abbreviated (e.g., *E. coli* for *Escherichia coli*). The data vividly demonstrate that the dominant IAI pathogens vary significantly across the globe, challenging a “one-size-fits-all” approach to treatment and prevention. While Gram-negative bacteria are the main driver of mortality worldwide, with *E. coli* leading in most regions, the emergence of *Staphylococcus aureus* as a key pathogen in high-income regions is a critical distinction. This regional heterogeneity underscores the necessity of tailoring antimicrobial stewardship and empirical antibiotic choices to local epidemiological data.

In 2021, *Escherichia coli* was the leading cause of IAIs-related deaths globally, responsible for an estimated 81 634 (73 626–89 641) deaths, corresponding to a death rate of 1.03 (0.93–1.14) per 100 000 population. *Klebsiella pneumoniae, Pseudomonas aeruginosa, S. aureus*, and *E. faecium* were followed as the next most common pathogens. Regionally, *E. coli* ranked first in most super-regions, with the highest death rate observed in Central Europe, Eastern Europe, and Central Asia at 2.05 (1.86–2.24) per 100 000. In sub-Saharan Africa and South Asia, *K. pneumoniae* posed a comparable burden to *E. coli*, with nearly overlapping death rates. Notably, in high-income regions, *S. aureus* surpassed *E. coli* to become the leading pathogen, with a death rate of 1.13 (1.02–1.25) per 100 000. Across all regions, *P. aeruginosa* and *E. faecium* consistently ranked among the top five contributors to mortality. *Candida* spp. was also a notable pathogen, ranking 6th to 7th in death numbers in most super-regions, but falling to 9th position in high-income regions. Importantly, deaths caused by Gram-negative rod infections remained the predominant contributor to IAIs’ mortality in every GBD super-region (Fig. 3, Supplemental Digital Content Figure S6, available at: http://links.lww.com/JS9/G517). National-level comparisons further highlight substantial heterogeneity: *E. coli* consistently ranked first in most low- and middle-income countries (e.g., Afghanistan, Angola, and Bangladesh), whereas *S. aureus* dominated in select high-income or microstate settings (Andorra, Australia, and Austria) (Supplemental Digital Content Table S3, available at: http://links.lww.com/JS9/G517). DALY patterns were similar, with *E. coli* leading globally, *S. aureus* exceeding *E. coli* in high-income regions, and *K. pneumoniae* ranking first in sub-Saharan Africa (Supplemental Digital Content Figure S6, available at: http://links.lww.com/JS9/G517).

### Burden of antimicrobial-resistant bacteria in IAIs

**Figure 4.**
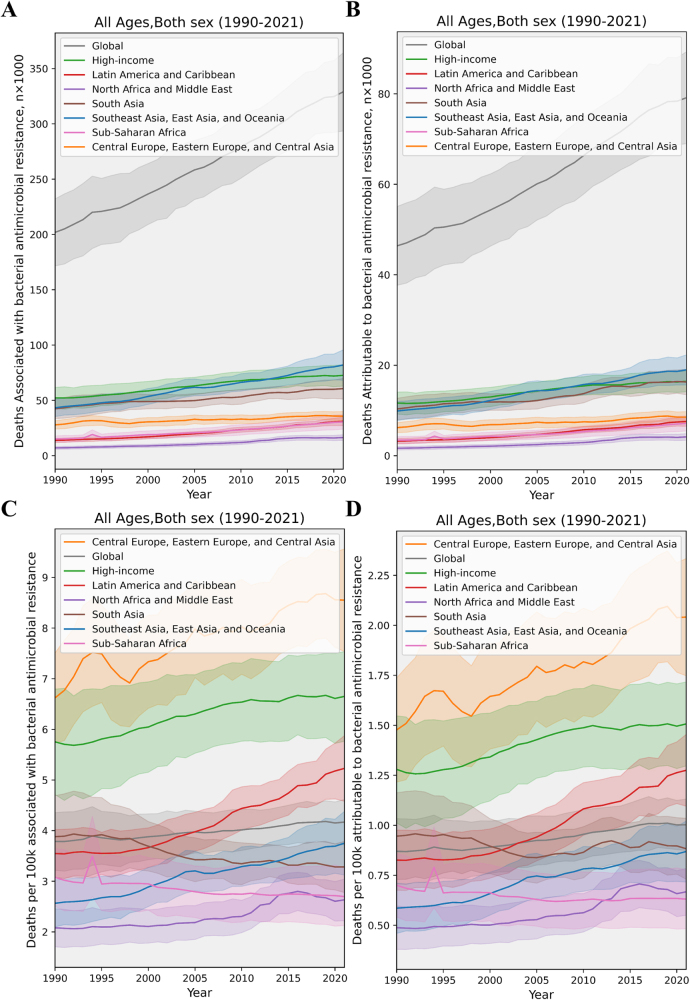
Trends in deaths associated with and attributable to antimicrobial resistance (AMR) in bacterial intra-abdominal infections, globally and by GBD super-region, 1990–2021. This figure tracks the mortality burden of AMR in IAIs from 1990 to 2021. Panels distinguish between deaths associated with AMR (occurring in patients with a resistant infection) and deaths attributable to AMR (excess deaths directly caused by resistance). Data are shown globally and by GBD super-region as (A, B) absolute numbers and (C, D) rates per 100 000 population. Shaded areas represent 95% uncertainty intervals. The graph highlights the escalating crisis of AMR: both the number of deaths and the death rates linked to resistant IAIs have steadily increased over the past three decades. The highest AMR-associated death rates are concentrated in Central/Eastern Europe and Central Asia. This rising trend signifies that AMR is not just a future threat but a present and growing cause of mortality, making AMR mitigation a critical component of improving surgical outcomes.

The number of global deaths associated with IAIs caused by antimicrobial-resistant bacteria rose from about 201 929 in 1990 to an estimated 328 929 in 2021. Over the same period, the global all-ages-associated death rate increased from 3.79 per 100 000 to 4.17 per 100 000. The highest associated rates were observed in Central Europe, Eastern Europe, and Central Asia, rising from 6.62 to 8.56 per 100 000. High-income regions also showed an increase from 5.75 to 6.65 per 100 000. Sub-Saharan Africa and South Asia experienced a slight decline (Fig. 4). Globally, DALYs associated with antimicrobial-resistant IAIs increased from 6.29 million in 1990 to 9.63 million in 2021, and the trend in DALY rates was generally consistent with that of death rates (Supplemental Digital Content Figure S7, available at: http://links.lww.com/JS9/G517).

### Burden of various drug-resistant pathogens in IAIs

**Figure 5.**
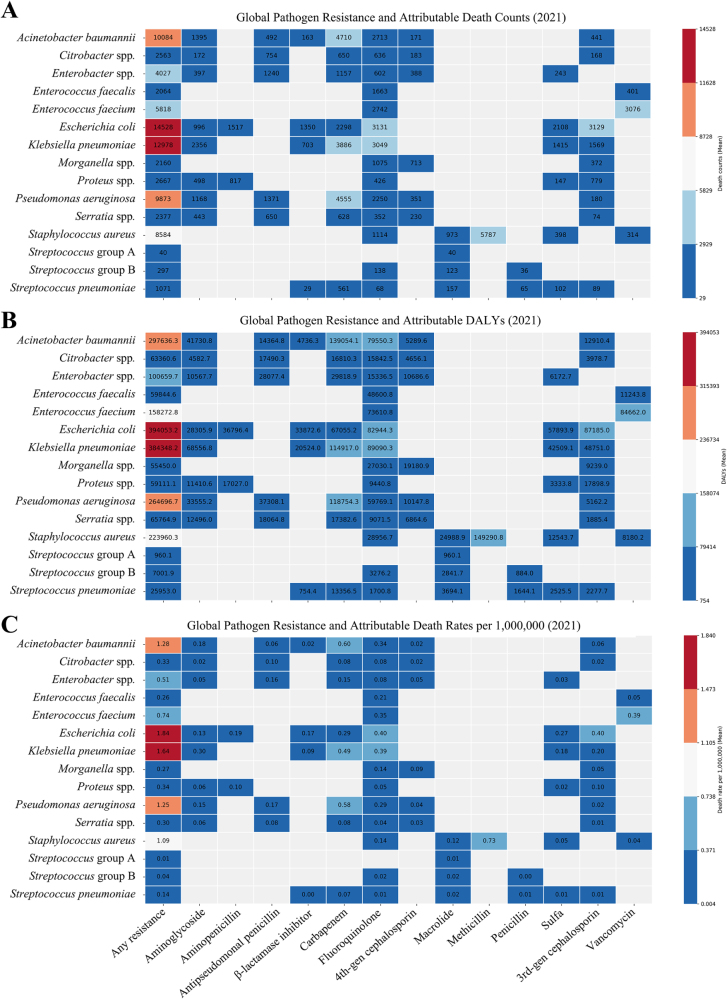
Global burden of pathogen-specific antimicrobial resistance attributable to antibiotic classes in 2021. This figure breaks down the global mortality and disability burden of AMR in 2021 by specific “bug-drug” combinations. Panels show the burden attributable to AMR, measured as (A) number of deaths, (B) disability-adjusted life years (DALYs), and (C) death rates. This detailed analysis pinpoints the most dangerous resistant pathogens in IAIs. Gram-negative bacteria are responsible for the vast majority (77.4%) of the AMR-attributable death burden. Specifically, carbapenem-resistant *Acinetobacter baumannii* and fluoroquinolone-resistant *Escherichia coli* are top contributors. Among Gram-positive bacteria, methicillin-resistant *Staphylococcus aureus* (MRSA) remains a major threat. This information is crucial for prioritizing drug development, diagnostics, and targeted stewardship efforts.

**Figure 6.**
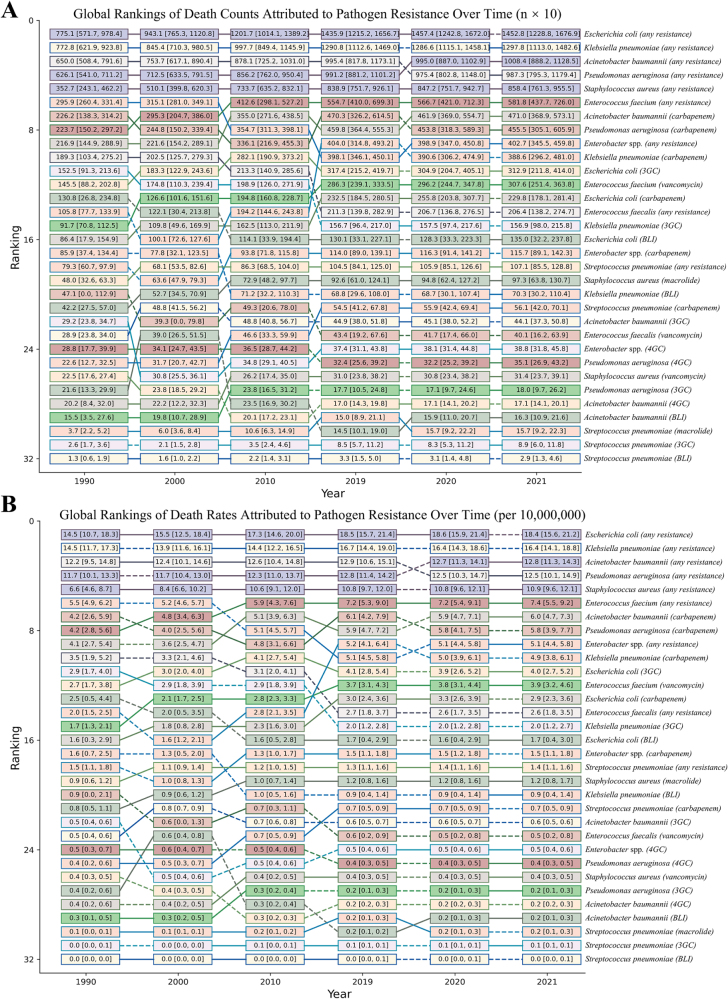
Global rankings of death burden attributed to pathogen resistance over time, 1990–2021. This figure visualizes the changing threat landscape of AMR by ranking the death burden attributable to specific pathogen-resistance combinations over three decades. Panels display the global rank of (A) attributable death counts and (B) attributable death rates. Lines track each pathogen’s trajectory. The rankings reveal a dynamic and evolving AMR threat. While resistance in *Escherichia coli* has consistently been the top-ranked problem, the most alarming trend is the rapid ascent of carbapenem-resistant pathogens, particularly carbapenem-resistant *Klebsiella pneumoniae* (CRKP) and *Acinetobacter baumannii*, in the global mortality rankings. In contrast, the rank of methicillin-resistant *Staphylococcus aureus* (MRSA) has remained relatively stable, possibly reflecting the success of targeted control measures in some regions. This highlights a critical shift in the AMR landscape toward multi-drug resistant Gram-negative bacteria.

In 2021, bacterial IAIs resistant to at least one antimicrobial class caused significant global mortality. *E. coli* was the leading pathogen, attributable to 14 529 deaths (1.84 per million). For specific resistance types, fluoroquinolone-resistant *E. coli* was attributable for 3131 deaths, CRKP attributable for 3886 deaths, carbapenem-resistant *P. aeruginosa* attributable for 4555 deaths, carbapenem-resistant *A. baumannii* attributable for 4710 deaths, and Methicillin-resistant *S. aureus* (MRSA) attributable for 5787 deaths per year (Fig. 5). Overall, Gram-negative bacteria accounted for about 61 257 attributable deaths (77.4% of the total), while Gram-positive bacteria caused 17 874 attributable deaths (22.6%). Associated death estimates were four to five times higher than attributable deaths but had a similar pathogen and resistance ranking (Supplemental Digital Content Figure S8, available at: http://links.lww.com/JS9/G517). The attributable DALY distribution was also similar, with *E. coli, K. pneumoniae*, and *A. baumannii* resistant to at least one antimicrobial among the main contributors (Fig. 5, Supplemental Digital Content Figure S8, available at: http://links.lww.com/JS9/G517). Between 1990 and 2021, attributable mortality, DALYs, and death rates from IAIs caused by antibiotic-resistant pathogens increased globally. *Escherichia coli* remained the leading pathogen, with attributable death rates rising from 14.5 to 18.4 per 10 million, followed by *K. pneumoniae, A. baumannii*, and *P. aeruginosa* (Fig. 6). Trends in associated deaths and death rates followed similar patterns but were approximately four to five times higher than attributable deaths and rates (Supplemental Digital Content Figure S9, available at: http://links.lww.com/JS9/G517). Attributable DALY trends closely mirrored these mortality patterns (Supplemental Digital Content Figure S10, available at: http://links.lww.com/JS9/G517). Death rates attributable to carbapenem-resistant *A. baumannii* increased from 4.2 to 6.0 per 10 million population, while those attributable to CRKP rose from 3.5 to 4.9 per 10 million. In contrast, MRSA and vancomycin-resistant *E. faecium* exhibited relatively stable attributable mortality patterns (Fig. 6).

Regional variations in pathogen-specific mortality were notable compared to global trends. In sub-Saharan Africa, *K. pneumoniae* surpassed *E. coli* as the top cause in 2021. South Asia remained dominated by *E. coli*, especially resistant strains. Southeast Asia and Oceania had a higher burden from *P. aeruginosa*. In high-income regions, Gram-positive pathogens, particularly MRSA and vancomycin-resistant *Enterococcus* (VRE), were most prevalent. Latin America and the Caribbean had a balanced burden from both Gram-negative and Gram-positive bacteria. CRKP was prominent in North Africa and the Middle East, while VRE ranked high in Eastern Europe and Central Asia (Supplemental Digital Content Figures S10–S15, available at: http://links.lww.com/JS9/G517).

## Discussion

Our analysis of the global burden of IAIs from 1990 to 2021 demonstrates a clear and concerning trajectory: overall mortality has risen significantly and is projected to remain a major public health challenge through 2050. This burden is not monolithic; it is characterized by profound regional variations in both the causative pathogens and the accelerating impact of AMR. While *E. coli* remains the leading pathogen globally, this is complicated by the emergence of *S. aureus* in high-income regions and, critically, by the divergent dynamics of specific resistant threats. Mortality from carbapenem-resistant *A. baumannii* and *K. pneumoniae* showed the most rapid increases, whereas MRSA-related mortality remained stable, and containment of vancomycin-resistant *Enterococcus faecium* proved less effective. These complex and varied trends underscore the need for nuanced, region-specific responses.

The rising incidence and mortality of IAIs appear to be driven by concurrent demographic and clinical trends. The overall upward trend in mortality may be partially driven by global population aging. For instance, a recent study has shown that the proportion of people aged ≥65 in China tripled between 1990 and 2020, reaching approximately 18% in 2020, a demographic shift expected to accelerate globally[17]. Consistently, our age-period cohort analysis demonstrated a marked increase in IAI-related mortality after the age of 65 years on a global scale (Supplemental Digital Content Figure S16, available at: http://links.lww.com/JS9/G517). Moreover, the global rise in surgical procedures, particularly among older adults, likely contributes to the increased incidence of postoperative IAIs[18]. This is clinically significant, as elderly patients undergoing abdominal surgery have a demonstrably higher risk of IAI-related mortality compared to younger patients^[19–22]^. In addition to demographic changes and surgical volume, other global trends may also have contributed to the observed patterns. The rising use of immunosuppressive therapies, including those for organ transplantation, cancer chemotherapy, and autoimmune diseases, may have increased susceptibility to IAIs in certain populations^[23,24]^. Indeed, the COVID-19 pandemic has further exacerbated these challenges. The disruptions in routine healthcare delivery, increased strain on surgical services, and delays in timely source control procedures have been well documented^[25,26]^. Recent pandemic-era data further demonstrate a measurable impact on AMR in IAIs. In China, a 6-year study of 2283 patients (5795 isolates) found *K. pneumoniae* as the predominant pathogen (17.9%), with carbapenem resistance reaching 75.9%, and imipenem resistance in *E. coli* rising from 13.8% to 25.1% during the pandemic (*P* < 0.001)[27]. Similarly, ICU surveillance data from Italy encompassing 299 000 admissions revealed that HAIs peaked during the pandemic years, with carbapenem-resistant Gram-negative bacteria contributing substantially to IAIs[28]. In Brazil, *P. mirabilis* exhibited increased resistance to all tested antimicrobials, alongside a higher prevalence of extended-spectrum β-lactamase-producing MDR strains between 2020 and 2022[29]. Furthermore, a meta-analysis reported a non-significant, yet clinically relevant increase in resistant Gram-negative organisms (IRR 1.64, 95% CI: 0.92–2.92), particularly in settings with suboptimal infection prevention and control measures and weak antimicrobial stewardship[30]. These findings highlight a pandemic-related surge in AMR and support recent guidelines calling for the incorporation of the up-to-date resistance data into IAI management and the reinforcement of antimicrobial stewardship programs[30].

Beyond demographic factors, regional differences in IAIs may reflect disparities in healthcare access, infection control, and antimicrobial stewardship. In sub-Saharan Africa, healthcare coverage remains critically low at 42.56%^[31,32]^. Most patients present late to hospital care, and microbiological diagnostic capacity is limited. Surveillance data from the World Health Organization (WHO) African Region (2016−2020) show low carbapenem resistance (0.1−2.5%) but a high rate of resistance to third-generation cephalosporins, including 31.5% for *E. coli*, 47.3% for *K. pneumoniae*, and 74.2% for *A. baumannii*^[33,34]^. These species are key pathogens in IAIs. Observed mortality patterns may therefore be driven as much by delayed access to care and inappropriate antibiotic use as by resistance itself^[35–37]^. In Eastern Europe, carbapenem-resistant *K. pneumoniae* is widespread, and nosocomial transmission has been well documented[38]. Approximately 75% of the AMR burden in this region is healthcare-associated[39]. Although the infection prevention and control programs could substantially reduce AMR and associated mortality, by 2022 some countries had still not fully implemented them[40]. In South Asia, all countries have developed national action plans for AMR; however, implementation remains weak. In 2018, phase ≥3 progress for AMR indicators was reported at 2.9% in Bangladesh, 16% in Nepal, 25% in India, and 26.6% in Bhutan[41]. Key drivers in this region include unregulated antibiotic sales, overuse in livestock, low awareness, and circulation of substandard drugs[42]. In East Asia, China has made notable progress in hospital-based antimicrobial stewardship, whereas improvements in the veterinary and agricultural sectors have been slower[43]. Uneven AMR mitigation across regions continues to influence the distribution of IAI pathogens and shape evolving resistance trends.

However, the global ecological design prevented the analysis of important patient- and procedure-related factors such as whether the operation was emergent or elective, timing from diagnosis to source control, and severity of illness before surgery. These variables influence postoperative outcomes and patterns of AMR. For example, emergency colorectal operations have consistently higher surgical site infection rates than elective cases, a difference reported in both single-center and multicenter studies^[44–46]^. IAI studies, including the AbSeS multinational cohort and work by Arvaniti *et al*[47], found that severe illness, diffuse peritonitis, and sepsis predict mortality, while shorter time to surgery improves survival[48]. Hospital resources and local resistance ecology may also confound results. Higher operative volume at both the surgeon and hospital levels is associated with lower mortality and fewer complications^[49,50]^. Intensive care unit occupancy has been linked to more healthcare-associated infections and transmission of multidrug-resistant organisms[51], with similar patterns observed in neonatal and pediatric care units. Outbreaks of carbapenem-resistant *A. baumannii* highlight the impact of unit design, nurse staffing, and surge conditions[52]. Preoperative carriage of extended-spectrum beta-lactamase-producing *Enterobacteriaceae* increases the risk of surgical site infections[53], and postoperative multidrug-resistant Gram-negative infections raise the mortality risk[54]. As these factors were not included in our dataset, residual confounding is probable, and observed regional differences should be considered associations rather than causal relationships.

Despite a notable overall increase in deaths from IAIs, regional disparities remain pronounced. For example, mortality surged in regions such as the high-income Asia Pacific and Southeast Asia, while it declined in South Asia and sub-Saharan Africa. The observed regional heterogeneity in mortality trends is likely multifactorial. In the high-income Asia Pacific and Southeast Asia, the surge in mortality may reflect a convergence of factors including rapidly aging populations, high rates of complex surgical interventions, dense urban healthcare settings with increased risk of hospital-acquired infections, and rising prevalence of multidrug-resistant Gram-negative pathogens^[2,13,55]^. Expanded access to surgical care in these regions over the last two decades, while beneficial, may paradoxically increase exposure to procedures and devices associated with IAIs, particularly in the absence of uniformly robust infection prevention infrastructure[56]. In contrast, the declines seen in South Asia and sub-Saharan Africa could in part be related to improvements in basic surgical safety protocols, gradual expansion of essential infection control measures, and targeted antibiotic stewardship campaigns supported by international health initiatives^[57,58]^. Additionally, in some lower resource settings, underdiagnosis or limited microbiological confirmation may lead to an underestimation of pathogen-specific mortality, contributing to the apparent downward trend[59]. These trends highlight that differences in health system capacity, antimicrobial stewardship implementation, and surveillance quality can substantially influence the epidemiology of IAIs across regions. Country-level outliers, such as Lithuania and Monaco, warrant cautious interpretation. In parts of Eastern Europe, high and, in some settings, increasing burdens of carbapenem-resistant Gram-negative pathogens, including *K. pneumoniae, E. coli, Enterobacter cloacae* complex, *A. baumannii*, and *P. aeruginosa*, have been associated with excess mortality in severe infections and may partly underlie higher rates of IAI-related deaths^[60–62]^. By contrast, Monaco’s markedly old age structure and the denominator effects inherent to its status as a microstate can inflate observed rates and widen 95% UIs, while differences in surveillance, coding, and pathogen ascertainment may accentuate apparent between-country contrasts^[61,63]^.

This study also found that the composition and distribution of causative pathogens varied significantly across regions. Globally, *E. coli* remained the leading pathogen associated with IAIs; it is important to note that across all infection types, based on the global burden analysis for 2019, *E. coli* was the leading pathogen for deaths directly attributable to bacterial AMR, accounting for an estimated 219 000 deaths. It was followed by *K. pneumoniae* (193 000 deaths) and *S. aureus* (178 000 deaths)[16]. However, in high-income regions, our IAI-specific analysis revealed a notable shift, with *S. aureus* emerging as the second most common pathogen. This finding is consistent with WHO reports identifying MRSA as a leading driver of AMR burden in Europe[64]. In sub-Saharan Africa and South Asia, *K. pneumoniae* was nearly as prevalent as *E. coli*, a pattern that aligns with regional AMR burden analyses from the WHO[33]. The regional prominence of other pathogens, such as *A. baumannii* and *Candida* spp., particularly in healthcare-associated contexts, further underscores geographic variations in epidemiology. The prevalence of *A. baumannii* in North Africa, the Middle East, and South Asia reflects challenges with nosocomial outbreaks and environmental persistence^[65–67]^, while the burden of invasive candidiasis is often higher in regions with limited healthcare resources, where delayed diagnosis contributes to severe outcomes^[68,69]^.

Another central finding of our study is the alarming rise in AMR-related IAI mortality, which increased from 201 929 deaths in 1990 to 328 929 in 2021. This trend is consistent with accumulating evidence indicating a worsening global burden of AMR. A 2019 WHO survey found that, of 129 000 intra-abdominal infection deaths in Africa, 82% were linked to resistance[33]. Among the resistant pathogens, Gram-negative bacteria, particularly Enterobacterales, represent the greatest challenge. Deaths linked to resistant *E. coli* strains increased from 40 154 to 68 247, while those from resistant *K. pneumoniae* rose from 32 824 to 46 640 during the study period. The sharp increase in fluoroquinolone-resistant *E. coli* mortality (from 12 084 to 45 559 deaths), together with the emergence of carbapenem-resistant *E. coli* and CRKP as major threats in North Africa, the Middle East, Latin America, and parts of Europe, correlates strongly with regional patterns of antibiotic consumption^[70–72]^. For instance, a landmark study tracking global antibiotic use from 2000 to 2015 reported a 65% increase in total consumption, from 21.1 to 34.8 billion defined daily doses, and a 39% rise in per capita use. This escalation is driven almost entirely by low- and middle-income countries, where regulatory oversight and stewardship are often weaker[73]. This trend has continued, with recent WHO GLASS reports documenting a 46% global increase in antibiotic consumption from 2000 to 2018, paralleled by rising resistance rates in critical pathogens like *K. pneumoniae* (35% carbapenem resistance globally) and *E. coli* (20–80% fluoroquinolone resistance across regions)[74]. Another strong ecological evidence confirms this association: a 2023 meta-analysis of 73 countries demonstrated that every 10% increase in antibiotic consumption correlated with a 4.3% rise in AMR prevalence (95% CI 2.2–6.5%) for key drug–bug combinations[75]. This quantitative link provides a strong explanatory basis for the geographic distribution of resistance observed in our analysis. The global spread of carbapenemase-producing strains (e.g., *K. pneumoniae* carbapenemase and *New Delhi metallo-β-lactamase*) has further accelerated this crisis, facilitated by factors like poor infection control and over-the-counter antibiotic sales^[72,76–78]^. Carbapenem-resistant *E. coli* has become particularly problematic in regions such as North Africa and the Middle East, consistent with previous findings[79]. Likely drivers include the widespread availability of carbapenems without prescription, weak antimicrobial stewardship programs, and fragmented healthcare delivery systems. *K. pneumoniae* resistant infections also showed worrisome trends. In regions such as Latin America, the Caribbean, Central and Eastern Europe, and Central Asia, CRKP emerged as a major threat. The findings support the prioritization of novel antibiotics targeting CRKP and extensively drug-resistant *A. baumannii.*

In contrast to the escalating threat from Gram-negative pathogens, the burden from Gram-positive resistant organisms like MRSA and VRE showed different dynamics. Between 1990 and 2021, MRSA-associated IAI deaths remained relatively stable globally, with the highest burden concentrated in high-income countries. This trend is consistent with reports showing that concerted infection control efforts, such as bundled interventions and mandatory screening in the US and Europe, successfully reduced MRSA incidence from peaks in the early 2000s^[76–78,80]^. Despite these successes, MRSA remains a formidable pathogen due to its environmental persistence and the emergence of virulent community-associated strains[81]. VRE-related deaths increased more modestly, a trend that aligns with previous reports^[82,83]^. The persistent challenge of VRE in hospital settings, especially among immunocompromised patients, is linked to its environmental resilience and the difficulty of controlling asymptomatic colonization^[84–86]^.

Our findings also have direct clinical implications for managing IAIs in regions with a high prevalence of AMR. In high-income regions where *S. aureus*, including methicillin-resistant strains, is a prominent pathogen, standard cephalosporin prophylaxis alone may be inadequate for certain patients. Current guidelines recommend perioperative vancomycin in addition to standard prophylaxis for those with documented colonization, a history of methicillin-resistant infection, or other recognized risk factors, while avoiding routine use in all patients to limit toxicity and resistance selection^[30,87,88]^. This risk-based approach ensures that empirical antibiotic choices reflect both local resistance surveillance and individual patient characteristics. The high mortality associated with resistant pathogens underscores the primacy of aggressive and timely surgical source control, which remains the definitive cornerstone of IAI management^[89–91]^. Effective elimination or drainage of the septic focus is often more decisive for survival than marginal differences between antimicrobial regimens. Intraoperative decisions should therefore focus on achieving adequate control at the initial operation. Minimally invasive procedures are preferred whenever feasible^[88,92]^. Observational data indicate that open colectomy carries approximately twice the risk of surgical site infection compared with laparoscopic colectomy, such as 7.9% versus 4.1% reported in a large cohort of colon surgeries^[93,94]^. Open laparotomy and reoperation have also been identified as independent risk factors for surgical site infection caused by MDROs[95]. In settings where such organisms are endemic, avoiding unnecessary reoperation, considering early image guided percutaneous drainage, and implementing comprehensive prevention bundles for unavoidable open procedures are essential strategies. These measures integrate surgical decision-making with antimicrobial stewardship, reducing unnecessary exposure to broad spectrum agents and improving outcomes.

Translating these clinical principles into policy and practice requires consideration of broader health system contexts, particularly the resource constraints and epidemiological patterns unique to different regions. In low- and middle-income countries, low-cost, high-impact measures such as the WHO Surgical Safety Checklist, timely narrow-spectrum prophylaxis, clipping rather than shaving hair, chlorhexidine–alcohol skin preparation, normothermia, and rapid protocolized source control effectively reduce surgical site and IAIs without requiring advanced laboratories[96]. A stepwise antimicrobial stewardship model using the WHO Access, Watch, Reserve classification framework, sentinel antibiograms, and simple audit-and-feedback is feasible where microbiology is limited[97]. In high-income countries, integrating rapid molecular diagnostics with stewardship is cost-effective, accelerates targeted therapy, and reduces mortality and hospital stay[98]. Barriers, including workforce capacity, sterile processing, and operating room workflow, can be addressed through phased roll-outs in high-burden hospitals, with indicators such as prophylaxis timeliness, time to source control, and 72-hour de-escalation. Embedding budget-impact and cost-effectiveness analyses within national AMR and surgical quality programs can enhance sustainability[97].

## Limitations

This study has several limitations. First, we excluded fungal pathogens to focus on bacterial AMR and because fungal surveillance data are sparse, inconsistent, and not comparable across regions. Second, our reliance on modeled data, particularly for regions with limited surveillance, introduces uncertainty and potential bias into our estimates. In many low- and middle-income countries, the lack of primary data required us to extrapolate from heterogeneous sources, which may impact data quality and the accuracy of resistance estimates. This underrepresentation of resistance data in resource-limited settings may lead to systematic underestimation of local AMR burdens and misclassification in global burden rankings, potentially masking regions with a disproportionately high true impact. Conversely, higher-quality and more abundant data from well-resourced areas may bias aggregated global estimates toward their epidemiological profile. In addition, several APC estimates exhibited wide UI or overlapped zero; such results were interpreted as inconclusive rather than definitive evidence of stability or change. This pattern often reflects underlying data sparsity, heterogeneous laboratory methods, and uneven surveillance quality. Accordingly, trends with wide intervals should be interpreted cautiously, recognizing that reduced precision may arise from variability in surveillance systems rather than actual epidemiological constancy. Furthermore, we were unable to distinguish community-acquired from hospital-acquired IAIs, which limits our ability to address important epidemiological differences between these groups. The focus on mortality and DALYs also prevented comprehensive assessment of incidence, prevalence, and the burden of non-fatal IAIs. Future efforts should prioritize strengthening surveillance systems and collecting more granular data to address these limitations. Finally, economic and operational factors, such as the cost-effectiveness, feasibility, and implementation challenges of recommended interventions, as well as variable access to essential antibiotics and diagnostics, were not explicitly incorporated into our analysis. These factors, along with macro-level changes in public health policy, resource allocation, and AMR stewardship program implementation, are likely to influence both incidence and outcomes, yet they lie beyond the scope of the current study design.

## Conclusion

This global analysis underscores the urgent need to address high IAI mortality alongside rising AMR, with strategies tailored to regional pathogen trends. In CRKP hotspots (e.g., North Africa, Middle East, and Latin America), key actions include restricting carbapenem use, adopting rapid resistance diagnostics, applying targeted novel agents, and strengthening environmental controls. In high-income regions with significant MRSA/VRE burdens, risk-based perioperative prophylaxis, targeted screening or decolonization, and strict contact precautions are critical. In resource-limited settings with high levels of Gram-negative AMR, priorities include establishing sentinel labs, using WHO Access, Watch, and Reserve–based antibiotic tiering, and implementing low-cost WHO surgical infection prevention bundles. Embedding measurable indicators, such as prophylaxis timeliness and carbapenem use rates, will facilitate monitoring and improvement of stewardship outcomes.

## Data Availability

The data used for the analyses in the study are publicly available at https://vizhub.healthdata.org/microbe/.

## Research registration unique identifying number (UIN)

This study is a secondary analysis of publicly available data from the MICROBE database. As it did not involve direct recruitment of, or interventions in, human participants, trial registration was not required.
